# Comparative efficacy of teriparatide and bisphosphonates or denosumab vs. teriparatide monotherapy in osteoporosis: a meta-analysis

**DOI:** 10.3389/fphar.2025.1605279

**Published:** 2025-05-21

**Authors:** Huan Jin, Cai Huang, Yan Zhang, Ying Dong, Qi Xiong, Di Wang, Ziyi He, Lin Shen, Chen Ma, Zixian Wang, Bo Shuai

**Affiliations:** ^1^ Department of Integrated Traditional Chinese and Western Medicine, Union Hospital, Tongji Medical College, Huazhong University of Science and Technology, Wuhan, China; ^2^ College of Sports Medicine, Wuhan Sports University, Wuhan, China; ^3^ Department of Pain, Union Hospital, Tongji Medical College, Huazhong University of Science and Technology, Wuhan, China; ^4^ Department of internal medicine, Rongjun Hostipal of Hubei, Wuhan, China

**Keywords:** osteoporosis, teriparatide monotherapy, bisphosphonates, denosumab, vertebral fractures, bone mineral density, bone turnover markers, meta-analysis

## Abstract

**Introduction:**

Teriparatide (TPTD), a widely used bone-promoting drug in osteoporosis (OP) treatment, may cause compensatory bone resorption with long-term monotherapy (>6 months). Combining TPTD with anti-bone resorption drugs (e.g., bisphosphonates and denosumab) could reduce bone loss, yet existing randomised controlled trials (RCTs) remain inconclusive regarding their effects on bone mineral density (BMD) and bone turnover markers (BTMs). This study aimed to systematically evaluate the effect of TPTD combined with bisphosphonates or denosumab on BMD and fracture risk in OP patients, compared with TPTD monotherapy.

**Methods:**

PubMed, Embase, Cochrane Library, and Web of Science databases (until March 2025) were searched for RCTs comparing TPTD monotherapy with combination therapy. Primary outcomes included vertebral/non-vertebral fracture risk reduction and BMD changes (lumbar spine, femoral neck, hip); secondary outcomes covered BTM variations and adverse events.

**Results:**

Eight RCTs (n = 787) were meta-analysed using Review Manager 5.4. Results showed: ① No significant differences in vertebral (OR = 0.93, 95%CI 0.12–6.93) or non-vertebral fractures (OR = 0.68, 0.31–1.46) between groups. ② TPTD combined with denosumab significantly increased lumbar spine (+3.40%, 0.44–6.36), femoral neck (+4.00%, 1.96–6.04), and hip BMD (+4.25%, 3.20–5.29). Bisphosphonate combinations improved hip BMD in the short term (<24 months: +1.81%, 0.65–2.97) but not long-term (≥24 months).③ Combination therapies regulated BTMs bidirectionally: bisphosphonates suppressed P1NP (40%–80% reduction vs. monotherapy), while denosumab preserved OC levels (-8–16% vs. monotherapy).④ Safety profiles were comparable: hypercalcemia incidence (16.3% vs. 14.7%, OR = 1.22, 0.55–2.69), musculoskeletal pain (9.8% overall), with no osteonecrosis cases reported.

**Conclusion:**

TPTD-denosumab combination is clinically preferable for BMD enhancement, though its long-term (>24 months) fracture risk reduction requires further validation.

## 1 Introduction

Osteoporosis is a metabolic bone disease characterised by osteopenia and bone microstructure degeneration. Approximately 30% of postmenopausal women and 20% of elderly men worldwide are affected by osteoporosis, while fracture-related disabilities and mortality rates remain high (Kanis et al., 2019; Lorentzon and Abrahamsen, 2022). Current treatment strategies include drugs that inhibit bone resorption (such as bisphosphonates and denosumab) and those that promote bone formation (such as TPTD). TPTD, a synthetic parathyroid hormone analogue, significantly increases bone mineral density (BMD) and reduces vertebral fracture risks by activating cyclic adenosine monophosphate (cAMP)/protein kinase A (PKA) signaling through intermittent binding to parathyroid hormone one receptors (PTH1R) on osteoblasts, thereby stimulating bone formation (Kondo et al., 2002). This anabolic effect is mechanistically complemented by denosumab’s targeted inhibition of the RANKL pathway: by binding to RANKL, denosumab prevents its interaction with RANK receptors on osteoclast precursors, thereby suppressing osteoclast differentiation and bone resorption (Lacey et al., 2012). Preclinical studies suggest that TPTD may further amplify this synergy by downregulating sclerostin expression, which enhances Wnt/β-catenin signaling to promote osteoblast activity while RANKL inhibition sustains anti-resorptive effects (Cheng et al., 2022; Tsai et al., 2013).

The limitations of BMD monotherapy have gradually emerged; long-term use may cause compensatory enhancement of bone resorption, and its effects diminish upon withdrawal due to rebound loss (Leder et al., 2015; Neer et al., 2001).

To overcome monotherapy limitations, combination or sequential regimens are increasingly adopted in clinical practice. Retrospective analyses indicate that 12%–18% of TPTD-treated patients transition to anti-resorptive agents (e.g., bisphosphonates or denosumab) after 12 months, predominantly through sequential protocols (6–12 months TPTD followed by bisphosphonates) or concurrent denosumab coadministration (Kocjan et al., 2021; Shane et al., 2022). Theoretically, these clinically prevalent strategies may produce a synergistic effect by coupling the bone formation-promoting effects of TPTD with osteoclastic inhibition. However, real-world implementation remains controversial—existing randomised controlled trials (RCTs) for combination regimens show inconclusive efficacy (Tsai et al., 2013; Anastasilakis et al., 2020; Ayub et al., 2021; Finkelstein et al., 2009). While bisphosphonates are historically preferred, recent guidelines conditionally recommend denosumab for high-risk patients due to superior BMD gains (Grade 2B) and fracture prevention potential (Morin et al., 2023).

Moreover, dynamic changes in bone turnover markers, such as serum procollagen type I amino-terminal propeptide (P1NP), osteocalcin (OC), and bone resorption markers such as collagen type I carboxy-terminal peptide (CTX) are key indicators to evaluate the early treatment response and underlying mechanism, yet their regulation patterns under different combination protocols require systematic integration (Eastell and Szulc, 2017; Vasikaran et al., 2011).

Although Meta-analyses have been conducted to explore TPTD efficacy, most studies have focused on single agents compared with other bone formation-promoting agents or only on a certain combination regimen (such as TPTD + bisphosphonates). Critical gaps persist in direct comparisons between bisphosphonates and denosumab when combined with TPTD, particularly regarding long-term (>24 months) outcomes and fracture risk reduction (Shen et al., 2017). In addition, previous reviews paid more attention to hard endpoints, such as fracture incidence, while giving limited attention to the dynamic changes in BMD and bone turnover markers, which can provide an early basis for clinical adjustment of treatment plans (Langdahl et al., 2018). Therefore, this study comprehensively evaluated the effect of TPTD combined with bisphosphonates or denosumab compared with monotherapy on BMD and bone turnover markers in patients with osteoporosis through a systematic search and meta-analysis, to provide evidence-based support for the optimal selection of combination drugs.

## 2 Materials and methods

### 2.1 Registration

The meta-analysis protocol in this study was registered with the Open Science Framework https://archive.org/details/osf-registrations-xuye5-v1 (registration number: https://doi.org/10.17605/OSF.IO/XUYE5). We performed this meta-analysis using A Measurement Tool to Assess Systematic Reviews.

### 2.2 Database literature search

This study strictly followed the process of the Specification for Systematic Reviews and Meta-Analysis Reporting and systematically searched the PubMed, Embase, Cochrane Library, Web of Science, and Google Scholar databases to obtain relevant literature. The retrieval strategy combined medical subject words (MeSH) and free words to ensure comprehensiveness and accuracy of the retrieval. Taking PubMed as an example, the search formula was designed as follows: (“teriparatide” [MeSH Terms] OR “teriparatide” [Title/Abstract] OR “TPTD” [Title/Abstract]) AND (“osteoporosis” [MeSH Terms] OR “osteoporosis” [Title/Abstract]) AND (“antiresorptive agents” [MeSH Terms] OR “antiresorptive drugs” [Title/Abstract] OR “bisphosphonates” [MeSH Terms] OR “denosumab” [Title/Abstract]), The inclusion criteria were RCTs comparing the efficacy and safety of TPTD in combination with bisphosphonates or denosumab versus monotherapy for the treatment of osteoporosis. The main evaluation indices included BMD growth rate of the lumbar spine and femoral neck and changes in bone turnover markers. The secondary evaluation index was the incidence of adverse reactions. After systematic screening and evaluation, eligible high-quality RCTs were included in this meta-analysis.

### 2.3 Literature inclusion and exclusion criteria

The inclusion criteria used in this study include: ① Study type: RCT blinded to the study. ② Subjects: Adult patients (≥18 years old) diagnosed with primary osteoporosis (based on World Health Organization criteria: lumbar spine or hip BMD T value ≤-2.5 or accompanied by fragility fracture). Postmenopausal women, older men, and patients with glucocorticoid-induced osteoporosis were included. Intervention measures: The experimental group received TPTD combined with anti-bone resorption drugs (bisphosphonate or denosumab). Control group: TPTD monotherapy. Outcome measures: Percentage change in lumbar spine or hip BMD, with reporting of baseline and follow-up data required. Secondary outcomes: Changes in the serum levels of bone turnover markers (P1NP, OC, and CTX). ⑤ Follow-up time: at least 6 months or more follow-up data were included. Language restrictions: Only studies published in English were included.

Exclusion criteria used in this study include: ① Non-randomised controlled trials, such as observational studies, case reports, reviews, or conference abstracts. ② Study subjects were secondary osteoporosis, such as those caused by glucocorticoids, abnormal thyroid function, or bone metastases from malignant tumours. ③ The experimental group or the control group used other bone formation-promoting drugs (such as abalotide and romosumab) or non-specified anti-bone resorption drugs, such as oestrogen and selective oestrogen receptor modulators. ④ BMD or bone turnover marker data cannot be extracted or estimated, where only the P value but no specific value was reported. ⑤ Repeatedly published literature, in which only the most complete or latest version of data was included. ⑥ Non-English literature. ⑦ Animal experiments or *in vitro* studies.

The literature screening process used in this study includes: ① Preliminary acquisition of literature through database search and removal of duplicate literature studies. ② Screening of title and abstract, excluding studies that do not meet the inclusion criteria. ③ Reviewing the full text and further screening the literature that meets the inclusion criteria. ④ Data extraction and quality assessment, such as using the Cochrane bias risk assessment tool. ⑤ Application of strict inclusion and exclusion criteria to ensure the scientific rigour and reliability of Meta-analysis, and provision of high-quality evidence to support the optimisation of anti-osteoporosis treatment strategies.

### 2.4 Data extraction

Two researchers with relevant professional backgrounds independently extracted key information from the literature according to the research purpose and screening criteria, including the first author, publication year, sample size, intervention measures, and evaluation indicators of the research results. During the information extraction process, if there was any disagreement, a third senior researcher conducted an arbitration to ensure the accuracy and consistency of data extraction.

### 2.5 Literature quality evaluation

To ensure the reliability and scientific rigour of the meta-analysis results, the Cochrane risk-of-bias tool was used to evaluate the included RCT quality. The evaluation includes the following six aspects: ① Random sequence generation: to evaluate whether the study uses the correct randomisation method, such as computer random number generation or random number table, to assign subjects. ② Assignment hiding: to assess whether the randomisation protocol is fully hidden from the investigator and the subject (e.g., using centralised randomisation or sealed envelope method). Blinding of subjects and researchers: to assess whether the subjects and researchers were blinded to the study to reduce implementation bias. Incomplete data reporting: to evaluate whether the study reported data comprehensively, accounted for losses to follow-up or withdrawals, and employed intention analysis to deal with missing data. Selective reporting: to evaluate whether the study reported all preset outcome indicators to avoid selective reporting bias. ⑥ Other biases: to assess whether there are other potential biases, such as funding sources, and research design defects. Each evaluation content is judged according to “low risk of bias (+1 point)”, “high risk of bias (−1 point)” or “uncertain (0 point)”. According to the evaluation results of the risk of bias, the literature quality included in the study was divided into three grades: grade A (4-6 points), grade B (2-3 points), and grade C (<2 points). The impact of the risk of bias on the results was considered in the meta-analysis. High-quality studies were given higher weights to improve the reliability of the analytical results.

### 2.6 Statistical analysis

RevMan 5.4 software was used for meta-analysis. Continuous variables, such as BMD growth rate, the mean difference (MD) and its 95% confidence interval (CI) were used as the effect size. Bicategorical variables (e.g., fracture incidence and adverse reaction incidence), odds ratio (OR) and 95% CI were used as the effect size. Heterogeneity was assessed by the I^2^ test. If I^2^ < 50%, heterogeneity is considered low and the Fixed-effects Model is used for analysis. If I^2^ ≥ 50%, the heterogeneity is considered high, then the Random-effects Model is used for analysis, and the source of heterogeneity is further explored by subgroup analysis or sensitivity analysis. Publication bias was assessed visually using an inverted Funnel Plot. All statistical analyses were performed at P < 0.05, as differences were considered statistically significant.

## 3 Results

### 3.1 Literature screening process and results

**FIGURE 1:**
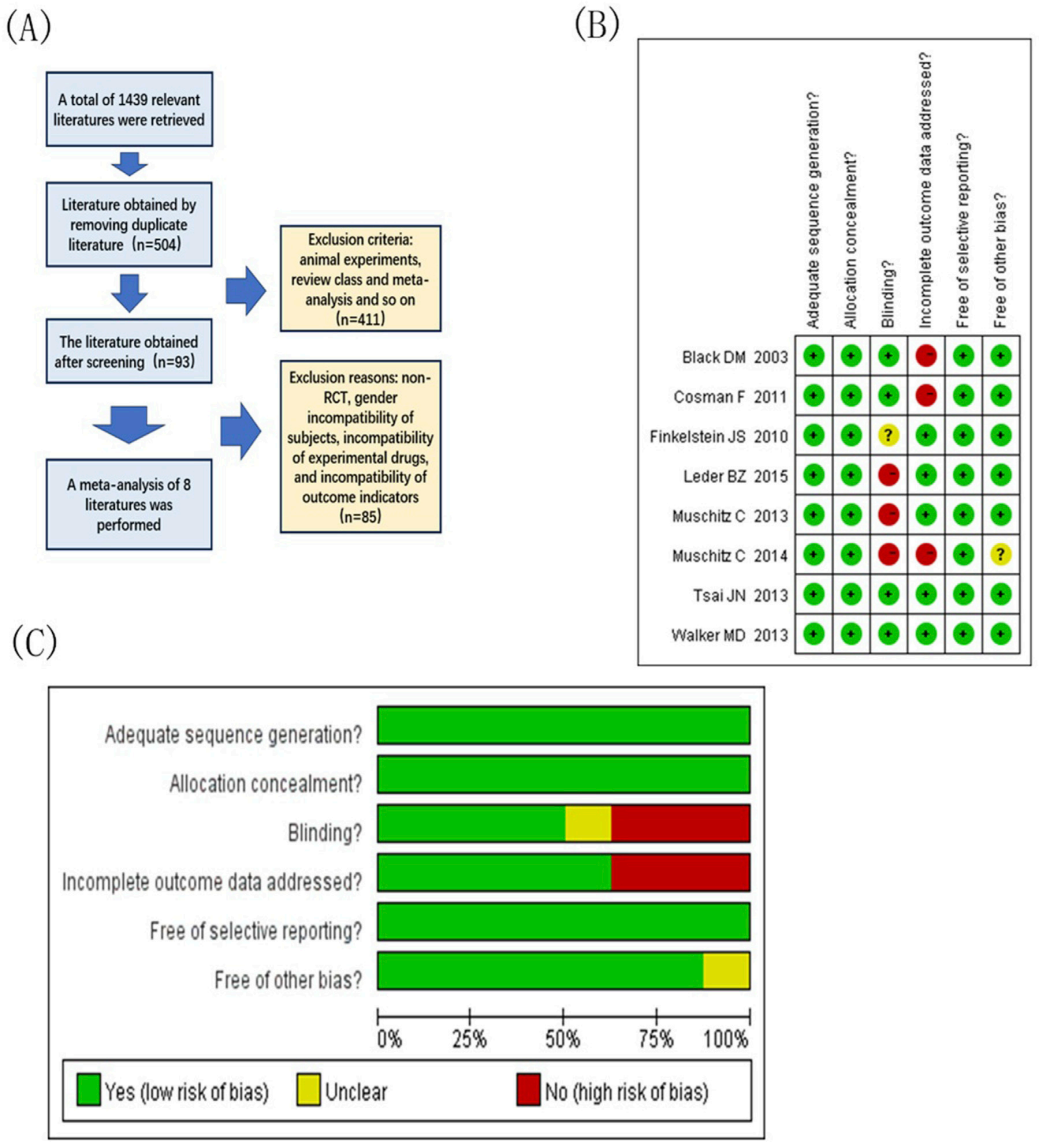
Document extraction and risk assessment map. **(A)** Literature screening flow chart; **(B)** Integrated migration map for quality assessment of included studies, “+” low-risk, “?” Unknown-risk, “-” high-risk; **(C)** Included study quality evaluation bias risk bar chat.

A total of 1,439 articles were searched in the English database, and 504 articles were obtained after eliminating unqualified articles using a computer. After reading the titles and abstracts, 411 studies were eliminated, including animal experiments, reviews, and meta-analyses, and 93 studies were included. After reviewing the full texts and excluding studies that did align with the subject’s situation, intervention indicators, or intervention measures, eight studies were finally included (Tsai et al., 2013; Cosman et al., 2011; Black et al., 2003; Finkelstein et al., 2010; Leder et al., 2014; Muschitz et al., 2014; Muschitz et al., 2013; Walker et al., 2013). A detailed screening diagram is presented in Figure 1A.

### 3.2 Basic characteristics of included studies

The eight included articles were RCTs published between 2003 and 2015. The population size ranged from nine to 150, with a total of 787 patients, excluding those lost to follow-up (TPTD + bisphosphonate or denosumab = 364; TPTD = 423). Of the included trials, two compared TPTD plus denosumab versus TPTD and six compared TPTD plus bisphosphonates versus TPTD alone. One study included male patients and the remaining seven studies included female patients. Specific information is provided in Table 1.

**TABLE 1:** General characteristics of patients included in the study.

Author	Country	Gender	EG	CG	Sample size (n = ?)		Age (X ± s)/year		Intervention		Basic treatment/day	Follow-up/month	Outcomes
					EG	CG	EG	CG	EG	CG			
Leder et al. (2015)	USA	F	Denosumab + TPTD	TPTD	28	24	65.9 ± 9.0	65.5 ± 7.9	Denosumab 60mg/6 months + TPTD 20ug/day, IM	TPTD 20ug/day, IM	Calcium Vit D	24	③④⑤
Walker et al. (2013)	USA	M	RID + TPTD	TPTD	10	9	56.7 ± 4.9	51.6 ± 3.9	RID 35 mg/week, oral + TPTD 20ug/day, IM	TPTD 20ug/day, IM	Calcium 500 mgVit D 400 IU	18	③④
Finkelstein et al. (2010)	USA	F	ALN + TPTD	TPTD	20	20	62 ± 7	65 ± 7	ALN 10 mg/day, oral + TPTD 100 μg/day, IM	TPTD 56.5 μg/week, IM	Calcium 1000–1200 mg Vit D 400 IU	30	①②③④⑤
Black et al. (2003)	USA	F	ALN + TPTD	TPTD	59	119	70.2 ± 6.8	69.4 ± 7.3	ALN 5 mg/day, oral + TPTD 56.6 μg/week, IM	TPTD 100 μg/day, IM	Calcium 500mgVitamin D 400 IU	12	③⑤
Muschitz et al. (2014)	Austria	F	ALN + TPTD	TPTD	41	47	72.4 ± 9.1	72.8 ± 8.9	ALN 70mg/weekoral + TPTD 20ug/day, IM	TPTD 20ug/day, IM	Calcium Vit D	12	④⑤
Cosman et al. (2011)	multi-center	F	ZOL + TPTD	TPTD	137	138	65 ± 8.8	63.8 ± 9.1	ZOL 5 mg/year Ivdrip + TPTD 20ug/day, IM	TPTD 20ug/day, IM	Calcium 1000–1200 mg Vit D 400–800 IU	12	①②
Muschitz et al. (2013)	Austria	F	ALN + TPTD	TPTD	39	37	71.6 ± 8.5	71.7 ± 9.3	ALN 70mg/weekoral + TPTD 20ug/day, IM	TPTD 20ug/day, IM	Calcium 1000mgVit D 800 IU	18	①②③④⑤
Tsai et al. (2013)	USA	F	Denosumab + TPTD	TPTD	30	29	65.9 ± 9.0	65.5 ± 7.9	Denosumab 60mg/6 months + TPTD 20ug/day, IM	TPTD 20ug/day, IM	Calcium 500 mgVit D 400 IU	12	③④⑤

Notes: F, female M, male; EG, experimental group CG, control group; TPTD, teriparatide; ALN, alendronate; RID, isedronic; ZOL, zoledronic; Vit D, vitamin D; IM, intramuscular injection.

### 3.3 Quality assessment results

Figures 1B, C show the overall risk of bias obtained after the quality assessment of the included studies. Among the studies, three were rated as low risk in the random assignment method, assignment hidden report, and selective outcome reporting field. In the area of outcome data integrity, five studies explicitly mentioned and rated as low risk. Among other excursion risks, seven studies were rated as low risk. In the blinded field, three studies are high risk. According to the literature quality evaluation criteria, the articles were divided into grades: three articles of Grade A, four articles of Grade B, and one article of Grade C.

### 3.4 Meta-analysis results

#### 3.4.1 Incidence of vertebral fractures

**FIGURE 2:**
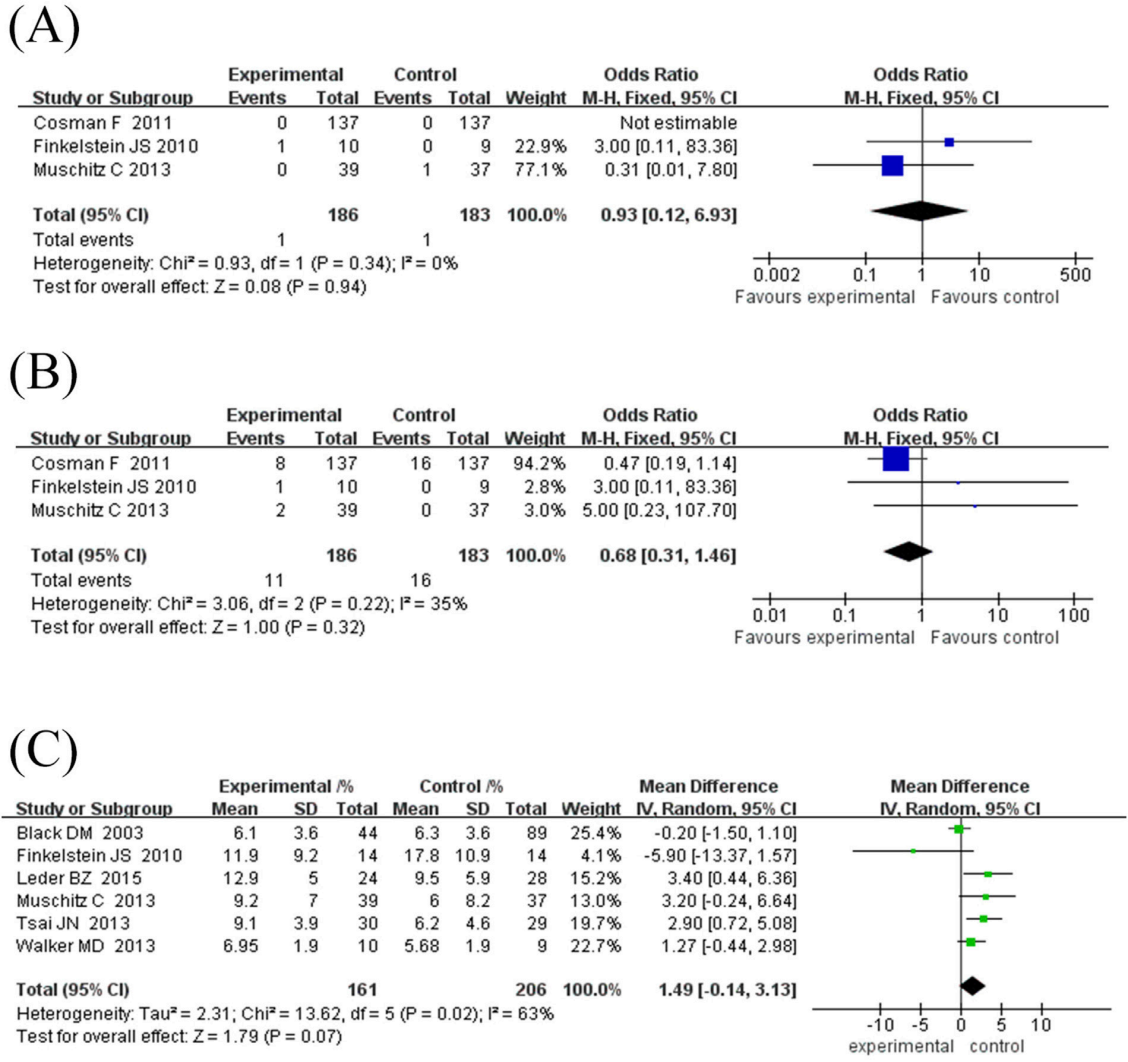
Forest map of fracture occurrence and growth rate of lumbar spine. **(A)** Forest map of vertebral fracture incidence (values <1 favor experimental group), **(B)** Forest map of non-vertebral fracture incidences (values <1 favor experimental group), **(C)** Forest map of lumbar spine bone mineral density (BMD) growth rate. Directionality note: Positive values indicate higher BMD gain in experimental group compared to control; The diamond/square position reflects the magnitude of BMD growth rate (left side represents smaller growth, right side represents larger growth).

This study included three randomised controlled trials (369 subjects) comparing the incidence of vertebral fractures between TPTD combined with anti-bone resorption drugs (test group) and TPTD alone (control group) (Figure 2A). Pooled analysis showed no significant difference in the incidence of vertebral fractures between the test and control groups (combined OR = 0.93, 95% CI [0.12, 6.93], Z = 0.08, P = 0.94). Specifically, no fracture events were observed in either group in the Cosman et al. (2011) study; One fracture occurred in the trial group of the Finkelstein et al. (2010) study (1/10) and no event occurred in the control group (0/9) (OR = 3.00, 95% CI [0.11, 83.36]); In the Muschitz et al. (2013) study, there was no event in the experimental group (0/39) and one fracture in the control group (1/37) (OR = 0.31, 95% CI [0.01, 7.80]). Although the heterogeneity test showed high agreement between the studies (I^2^ = 0%, P = 0.34), the confidence intervals were wide, indicating unstable results. Current evidence shows that combination therapy does not significantly reduce the risk of vertebral fractures. However, due to the extremely low incidence of events (one case in the total number of events in the test group and one case in the control group), the statistical efficacy is limited, and further verification is needed in a larger sample study.

#### 3.4.2 Incidence of non-vertebral fractures

This study included three randomised controlled trials (369 subjects) comparing the incidence of non-vertebral fractures between TPTD combined with anti-bone resorption agents (test group) and TPTD alone (control group) (Figure 2B). The pooled analysis showed no significant difference in the risk of nonvertebral fractures between the two groups (combined OR = 0.68, 95% CI [0.31, 1.46], Z = 1.00, P = 0.32). Specifically, eight fractures (8/137) occurred in the experimental group of the Cosman et al. (2011) study and 16 (16/137) in the control group (OR = 0.47, 95% CI [0.19, 1.14]). One fracture occurred in the experimental group of the Finkelstein et al. (2010) study (1/10) and no event occurred in the control group (0/9) (OR = 3.00, 95% CI [0.11, 83.36]). Two fractures occurred in the experimental group of the Muschitz et al. (2013) study (2/39) and no events in the control group (0/37) (OR = 5.00, 95% CI [0.23, 107.79]). There was mild heterogeneity among the studies (I^2^ = 35%, P = 0.22); however, the difference was not statistically significant. Notably, the low number of total non-vertebral fracture events (11 in the experimental group and 16 in the control group) may have contributed to insufficient statistical power. The current results suggest that combination therapy does not significantly reduce the risk of non-vertebral fractures; however, studies with larger sample sizes are required to confirm the observed differences in efficacy.

#### 3.4.3 Lumbar spine BMD growth rate

Six randomised controlled trials comprising 367 participants were included in this study to evaluate the effect of TPTD combined with anti-bone resorption drugs (experimental group) versus TPTD alone (control group) on the growth rate of lumbar BMD (Figure 2C). The overall analysis showed that the growth rate of lumbar BMD in the experimental group was significantly higher than that in the control group (combined MD = 1.49%, 95% CI [-0.14, 3.13], Z = 1.79, P = 0.07), but did not reach the statistical significance threshold; However, there was high heterogeneity between studies (I^2^ = 63%, P = 0.02), and further subgroup analysis using a fixed-effects model showed that the combination therapy significantly improved BMD (MD = 1.09%, 95% CI [0.23, 1.94], Z = 2.49, P = 0.01).

**FIGURE 3:**
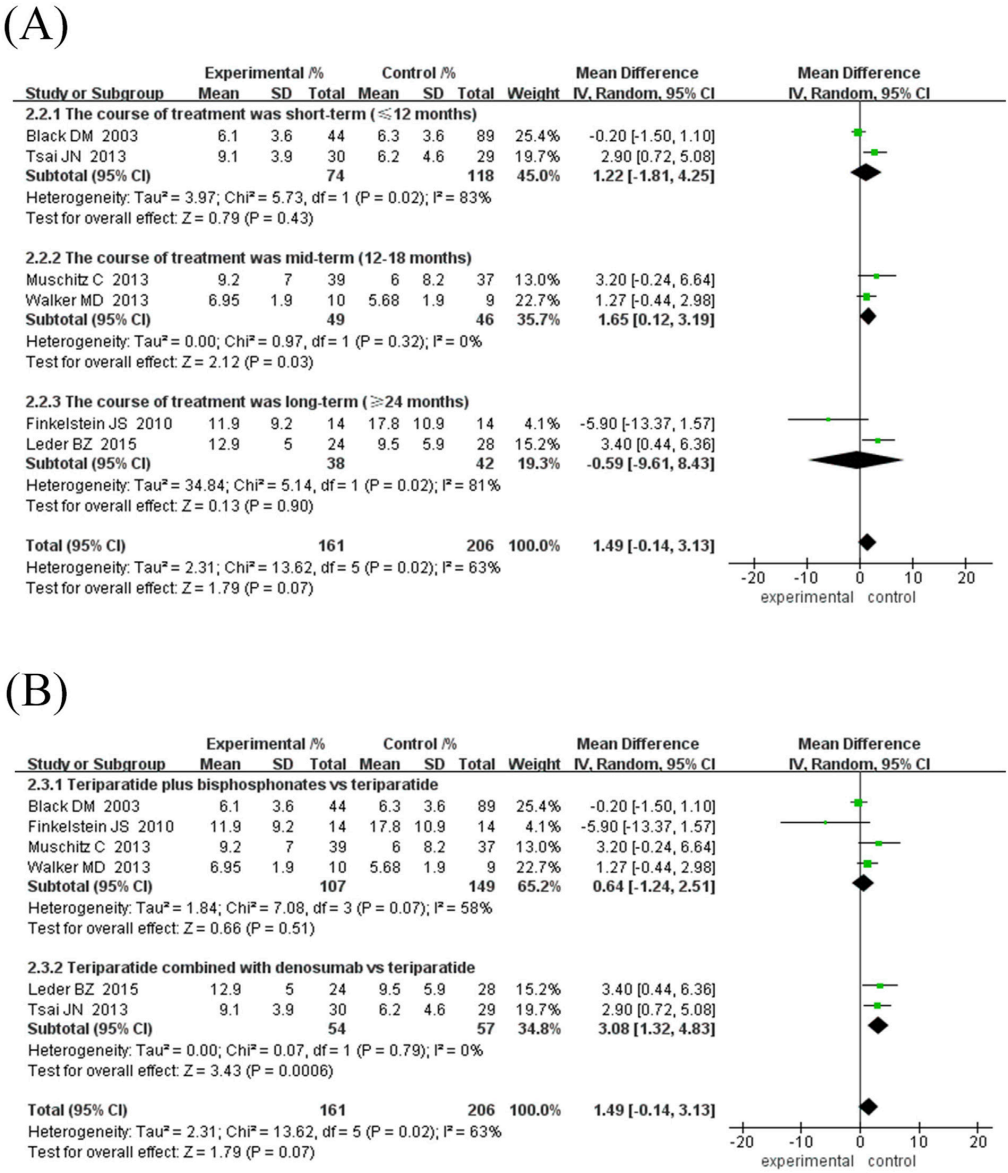
Forest map of BMD growth rate subgroup analysis in lumbar spine. **(A)** Subgroup analyses according to treatment duration, **(B)** Subgroup analysis based on antiresorptive drug types. Directionality note: Positive values indicate experimental group superiority; Marker position corresponds to growth magnitude (left = smaller, right = larger).

Subgroup analysis showed (Figures 3A, B): ① Difference in treatment course: There was no significant difference between the subgroup treated for 12 months (MD = 0.61%, 95% CI [-0.50, 1.73], Z = 1.08, P = 0.28) and the subgroup ≥24 months (MD = 2.14%, 95% CI [-0.62, 4.89], Z = 1.52, P = 0.13), while the subgroup treated for 18 months had a significant improvement in BMD (MD = 1.65%, 95% CI [0.12, 3.18], Z = 2.12, P = 0.03). ② Drug type: The subgroup combined with bisphosphonates did not show a significant increase in BMD (MD = 0.46%, 95% CI [-0.52, 1.45], Z = 0.93, P = 0.35); however, the subgroup combined with denosumab showed a significant increase (MD = 3.08%, 95% CI [1.32, 4.83], Z = 3.43, P = 0.0006).

In specific studies, Leder et al. (2015) (MD = 3.40%, 95% CI [0.44, 6.36]) and Tsai et al. (2013) (MD = 2.90%, 95% CI [0.72, 5.08]) showed obvious advantages of denosumab combination therapy, while Finkelstein et al. (2010) (MD = −5.90%, 95% CI [-13.37, 1.57]) suggested that long-term TPTD combination with bisphosphonate therapy may reduce BMD growth. There was high inter-study heterogeneity (I^2^ = 63%–83%), which may be related to differences in treatment regimens and durations. The current results show that TPTD combined with denosumab may be more effective in improving lumbar BMD. However, additional high-quality studies are needed to verify the differences in the efficacy of different drug types and treatment courses.

#### 3.4.4 Femoral neck bone density growth rate

**FIGURE 4:**
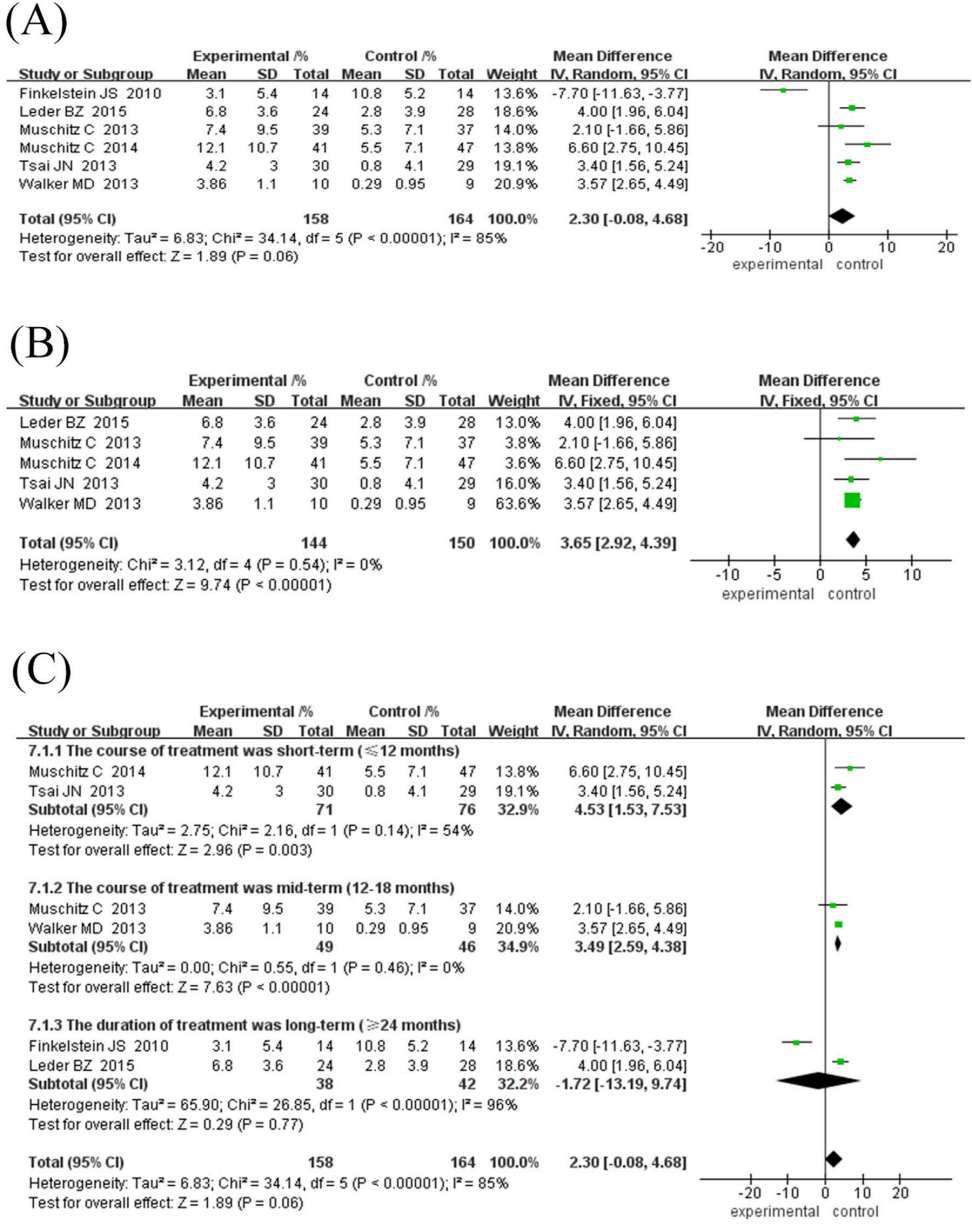
Forest map of BMD growth rate subgroup analysis in the neck of femur. **(A)** Forest map of BMD growth rate in the neck of femur, **(B)** Sensitivity analysis of forest map after removing Finkelstein JS’s study, **(C)** Subgroup analyses according to treatment duration. Directionality annotation: Rightward values denote greater BMD improvement in experimental group; Plot position indicates effect size magnitude.

Six randomised controlled trials comprising 322 participants were included to evaluate the effects of TPTD combined with anti-bone resorption drugs (experimental group) versus TPTD alone (control group) on lumbar BMD growth rate (Figure 4A). The overall analysis showed that the growth rate of lumbar BMD in the experimental group was significantly higher than that in the control group (MD = 2.30%, 95% CI [-0.08, 4.68], Z = 1.89, P = 0.06) but did not reach the statistical significance threshold; however, there was high heterogeneity among studies (I^2^ = 85%, P < 0.00001). The sensitivity analysis showed that only the Finkelstein et al. (2010) study course was 30 months, and the rest of the studies did not exceed 24 months. After excluding the study by Finkelstein et al. (2010), heterogeneity was significantly reduced (I2 decreased from 85% to 0% and P = 0.54).

Given the inconsistency between the existing course of treatment and the types of anti-bone-resorption drugs in the combination treatment group, a subgroup analysis was performed according to the course of treatment and types of anti-bone-resorption drugs. The results of both subgroup analyses showed that combination treatment significantly improved BMD (MD = 3.27%, 95% CI [2.55, 3.99], Z = 8.89, P = 0.10); however, the observed difference was not statistically significant.

**FIGURE 5:**
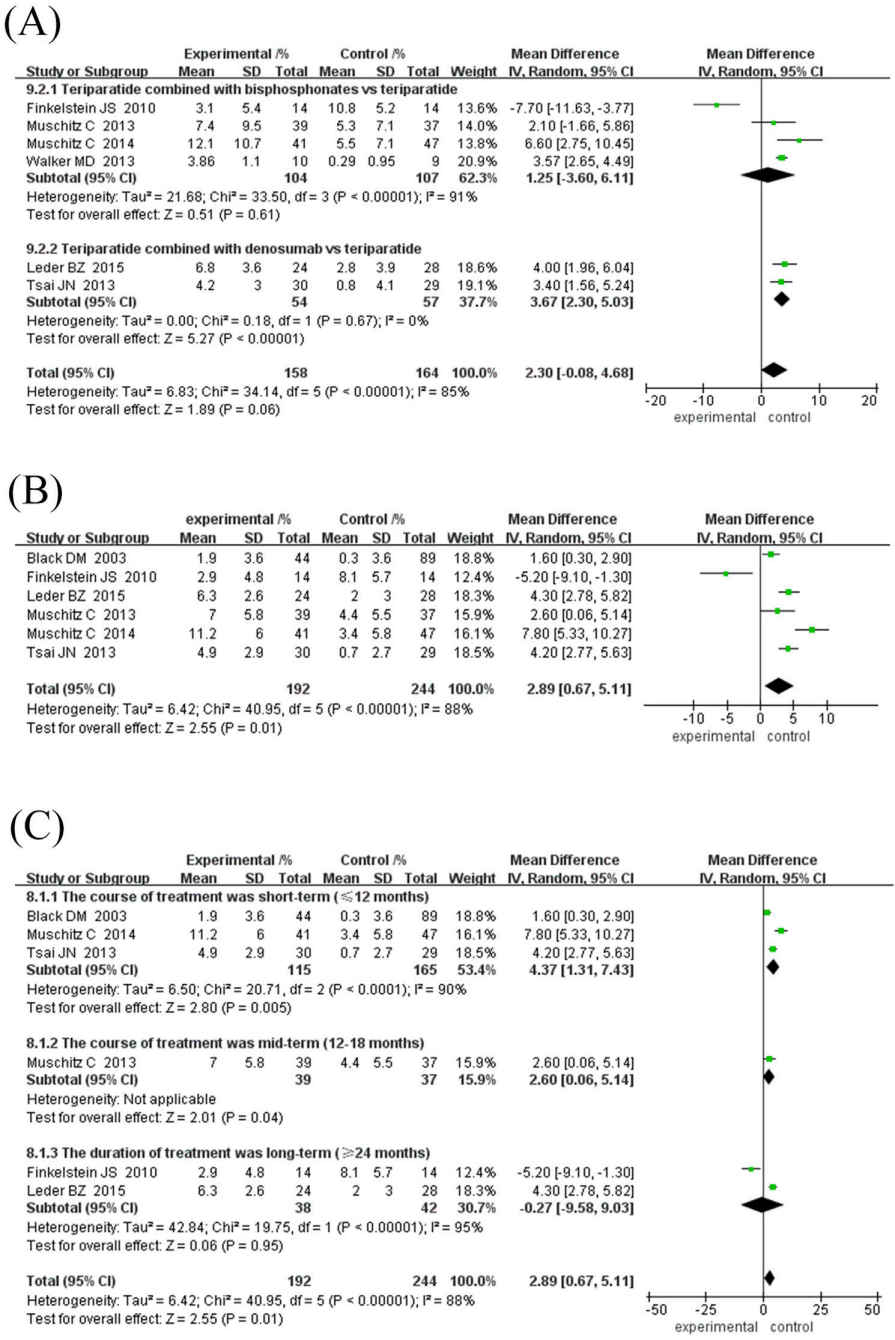
Forest plot of femoral neck BMD growth rate subgroup analysis and hip BMD growth rate. **(A)** Subgroup analysis of femoral neck BMD based on the type of antiresorptive drugs, **(B)** Forest map of the growth rate of neck of femur BMD, **(C)** Subgroup analysis of hip BMD based on treatment duration. Interpretation guidance: Positive standardized mean differences favor experimental intervention; Left-side positioning of markers indicates relatively smaller growth effects.

Subgroup analysis showed: ① Difference in treatment courses (Figure 4C): the subgroup treated for 12 months (MD = 3.99%, 95% CI [2.33, 5.65], Z = 4.72, P < 0.00001) and the subgroup treated for 18 months (MD = 3.49%, 95% CI [2.59, 4.38], Z = 7.63, P < 0.00001). The combination treatment group improved BMD significantly, while the treatment course ≥24 months subgroup (MD = 1.51%, 95% CI [-0.30, 3.32], Z = 1.64, P = 0.10). ② Drug type (Figure 5A): the subgroup combined with bisphosphonates (MD = 3.11%, 95% CI [2.26, 3.96], Z = 7.17, P < 0.00001) and the subgroup combined with denosumab. The effect was significant (MD = 3.67%, 95% CI [2.30, 5.03], Z = 8.87, P < 0.00001), as both groups significantly improved BMD.

In specific studies, Leder et al. (2015) (MD = 4.00%, 95% CI [1.96, 6.04]) and Tsai et al. (2013) (MD = 3.40%, 95% CI [1.56, 5.24]) showed obvious advantages in denosumab combination therapy, while Finkelstein et al. (2010) (MD = -7.70%, 95% CI [-11.63,-3.77]) suggested that long-term TPTD combined with bisphosphonate therapy may reduce BMD growth. Subgroup heterogeneity with treatment course ≥24 months (I^2^ = 96%, P < 0.00001) and the combined bisphosphonate group (I^2^ = 91%, P < 0.00001) is high, which may be related to the type of bisphosphonate and the specific treatment course. The current results indicate that TPTD combined with denosumab may be more effective in improving femoral neck BMD. However, more high-quality studies are needed to verify the differences in the efficacy of different drug types and courses of treatment.

#### 3.4.5 Hip joint BMD growth rate

Six studies (192 in the experimental group and 244 in the control group) were included in this meta-analysis to evaluate the effect of TPTD combined with anti-bone resorption drugs versus monotherapy on the growth rate of hip BMD (Figure 5B). The overall analysis showed that the bone density growth rate in the combination group was significantly higher than that in the single group (MD = 2.89%, 95% CI [0.67, 5.11], Z = 2.55, P = 0.01); however, the heterogeneity was higher (I^2^ = 88%, P < 0.00001).

**FIGURE 6:**
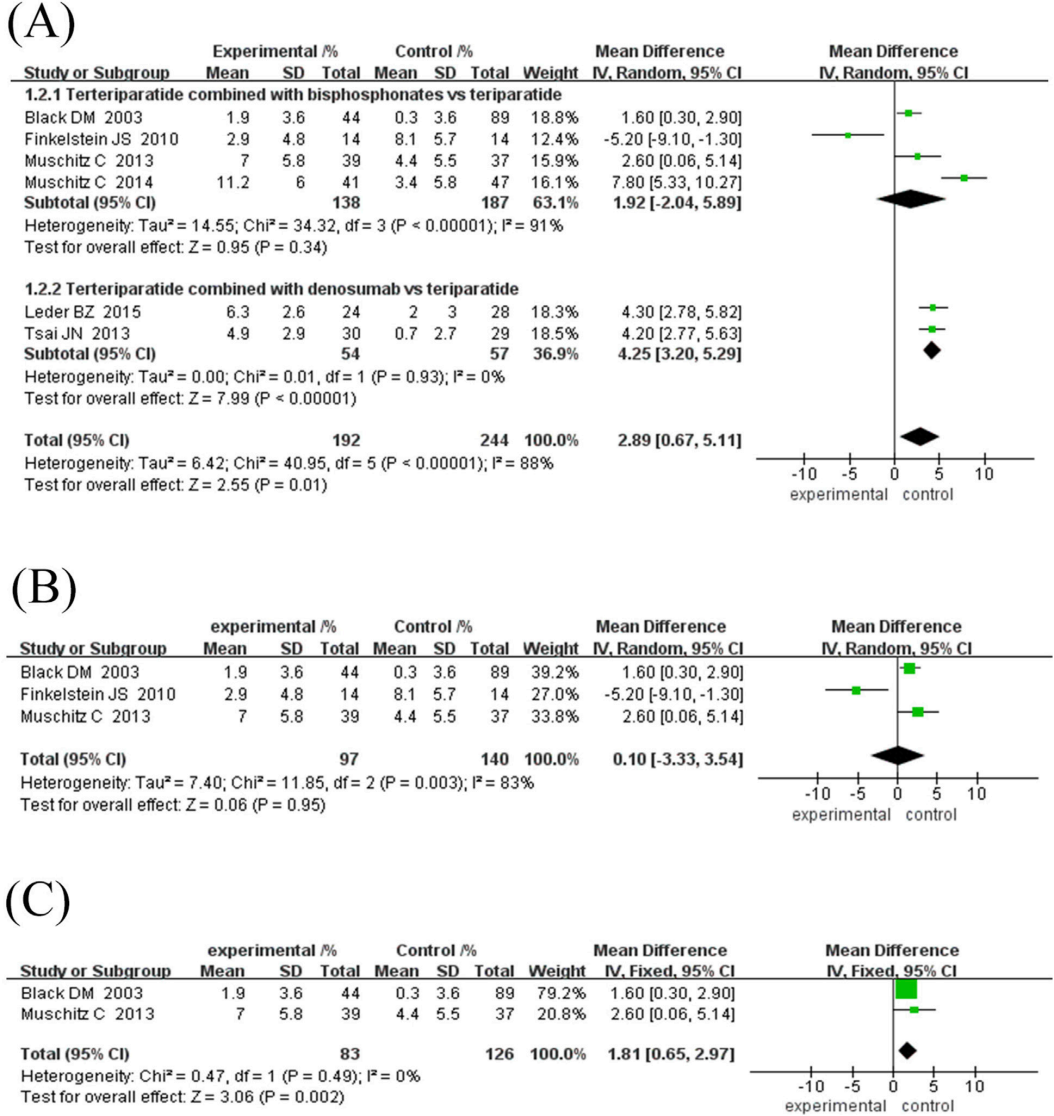
Forest plot of hip BMD subgroup analysis and sensitivity analysis. **(A)** Subgroup analysis of hip BMD based on the type of antiresorptive drugs, **(B)** Sensitivity analysis after removing Muschitz C’s study, **(C)** Sensitivity analysis after removing Finkelstein JS’s study. Directional reference: Right of unity line = experimental group advantage; Horizontal marker placement reflects effect size gradation.

Subgroup analysis showed that the efficacy varied depending on the course of treatment and the type of anti-bone resorption drugs: ① Treatment course subgroup (Figure 5C): the 12-month treatment group had the best effect (MD = 4.37%, 95% CI [1.31, 7.43], Z = 2.80, P = 0.006), followed by the 18-month group (MD = 2.60%, 95% CI [0.06, 5.14], Z = 2.01, P = 0.04), while the treatment course ≥24 months group had no significant difference (MD = -0.27%, 95% CI [-9.58, 9.03] However, the heterogeneity among the studies was high, and the 18-month treatment subgroup was only included in one study, resulting in insufficient statistical efficacy to draw a definitive conclusion. ② Subgroup of drug types (Figure 6A): The efficacy of the combined denomab group was significantly better than that of the single drug group (MD = 4.25%, 95% CI [3.20, 5.29], Z = 7.99, P < 0.00001, I^2^ = 0%), while the combined bisphosphonate group was effective (MD = 2.34%, 95% CI [1.33, 3.35], Z = 4.53, P < 0.00001), but the heterogeneity was extremely high (I^2^ = 91%, P < 0.00001).

Sensitivity analysis for the combination bisphosphonate subgroup showed (Figures 6B, C) that after excluding low-quality literature and studies with treatment duration ≥30 months, heterogeneity was eliminated (I^2^ = 0%, P = 0.49), and the efficacy remained significant (MD = 1.81%, 95% CI [0.65, 2.97], Z = 3.06, P = 0.002), suggesting that the original heterogeneity may be due to differences in study design and treatment duration.

In summary, combined denosumab or short-term bisphosphonate therapy can significantly increase hip BMD; however, more high-quality studies are needed to optimise the course of treatment and reduce heterogeneity.

#### 3.4.6 Changes in bone turnover markers

All eight RCTs included in this study reported dynamic changes in bone turnover markers, including bone formation markers such as P1NP, OC, andCTX and tartrate-resistant acid phosphatase 5b. However, quantitative meta-analysis could not be performed due to differences in data reporting forms—primarily descriptive trends or per-protocol analyses. Based on the results of various studies, the effects of combination therapy on bone metabolism show the following characteristics.

##### 3.4.6.1 Bidirectional modulation of bone formation markers

All studies consistently showed that TPTD alone significantly elevated bone formation markers (P1NP and OC), with peaks usually occurring at 6–12 months (e.g., 150–693% increase in P1NP), followed by a gradual decrease (Finkelstein et al., 2010; Cosman et al., 2011; Leder et al., 2015). However, the addition of anti-bone-resorption drugs significantly weakened the bone formation-promoting effect of TPTD. When combined with bisphosphonates (such as alendronic acid and zoledronic acid), the increase in P1NP and OC was 40%–80% lower than that in the single-agent group, and some studies even showed an early decrease (Tsai et al., 2013; Muschitz et al., 2013). However, when combined with denosumab, the inhibition of bone formation markers is relatively light. For example, the decrease in OC is 8%–16% lower than that of denosumab alone), suggesting that denosumab may retain part of its bone formation activity by targeting the Receptor Activator of Nuclear Factor-κ B Ligand (RANKL) mechanism (Leder et al., 2015; Tsai et al., 2013).

##### 3.4.6.2 Dynamic balance of bone resorption markers

The bone resorption markers (CTX and NTX) in the TPTD monotherapy group usually showed a delayed increase after the rise of bone formation markers (such as CTX peaks at 6–12 months), reflecting the bone-remodelling coupling effect (Finkelstein et al., 2010). Anti-bone-resorption drugs significantly inhibited bone resorption markers in the combination treatment group. CTX decreased by 50%–70% in the bisphosphonate combination group, showing a reduction comparable to bisphosphonate monotherapy. In contrast, CTX inhibition in the denosumab combination group was more durable (e.g., 57%–65% reduction), although the degree of inhibition after combination with TPTD was slightly weakened compared to denosumab alone (Leder et al., 2015; Muschitz et al., 2013). Notably, some studies have observed a transient increase in CTX in the early stage (first 3–6 months) of combination therapy, which may be related to the transient predominance of the pro-absorption TPTD effect (Tsai et al., 2013).

##### 3.4.6.3 Heterogeneous effects of drug type and treatment course–Bisphosphonates vs. denosumab

Bisphosphonates inhibited bone turnover markers more strongly and had a faster onset of action, whereas the denosumab combination group showed a more stable bone metabolic balance during long-term treatment (>12 months) (Leder et al., 2015; Muschitz et al., 2013).

##### 3.4.6.4 Treatment course dependence

When combined with bisphosphonates, the inhibitory effect of bone formation markers intensified with treatment course extension. For example, P1NP increased by only 107% compared with baseline at 30 months vs. 199% in the single-agent group, suggesting that long-term combination may lead to “excessive inhibition”. However, the denosumab combination group maintained moderate bone formation activity at 24 months (48% reduction in OC vs. 55% in the single-agent group) (Leder et al., 2015; Finkelstein et al., 2010; Tsai et al., 2013).

Association of marker changes with clinical endpoints Although combination therapy significantly modulates markers of bone turnover, its association with improved BMD or reduced risk of fracture has not been defined. For example, some studies have shown that although the combination of denosumab inhibits bone resorption, it still improves BMD through a synergistic mechanism. Although the bisphosphonate combination inhibits bone turnover more thoroughly, it may weaken the benefit of BMD in the long run (Tsai et al., 2013; Finkelstein et al., 2010).

In summary, TPTD combined with anti-bone-resorption drugs can affect bone turnover markers by regulating the two-way balance of bone metabolism; however, its effect varies with the drug type, course of treatment, and marker type. Future studies should standardise the reporting criteria for bone turnover markers (such as baseline values, absolute changes, and variability) to support quantitative synthetic analyses.

#### 3.4.7 Safety assessment

**FIGURE 7:**
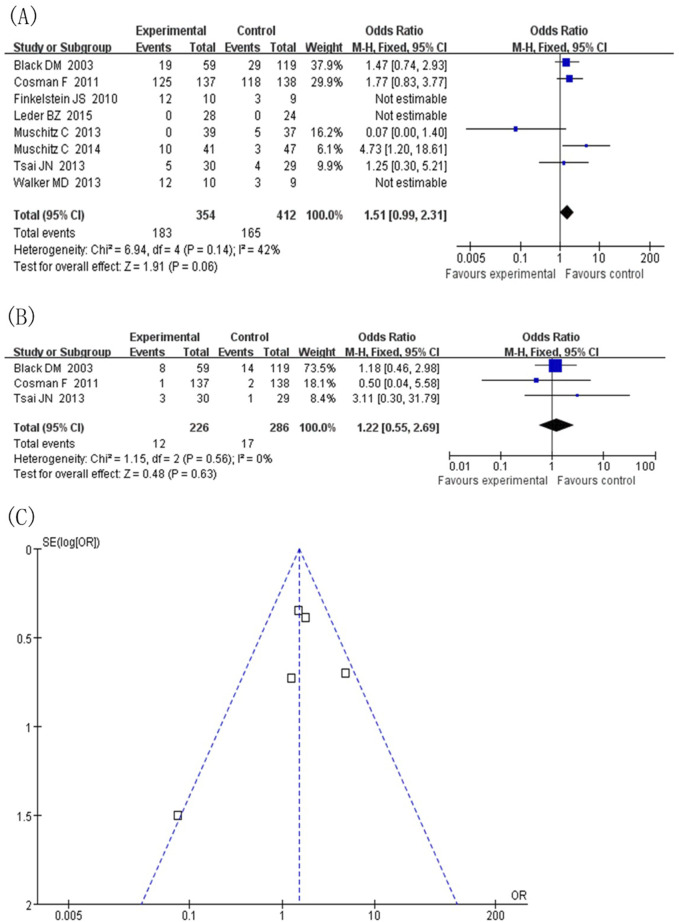
Adverse events. **(A)** Forest map of adverse events (values <1 favor experimental group), **(B)** Subgroup analysis of hypercalcemia, **(C)** Inverted funnel diagram of adverse event statistics.

Safety statistics were calculated for all included studies. In terms of total adverse events, comparing the experimental group (TPTD combined with anti-bone resorption drugs) with the control group (TPTD alone) (Figure 7A), the combined effect value was close to the statistical significance threshold (OR = 1.51, 95% CI [0.99, 2.31], Z = 1.91, P = 0.06), suggesting that the combination did not significantly increase the risk of total adverse events, and the heterogeneity was low (I^2^ = 42%, P = 0.14). Subgroup analysis of hypercalcaemia, the highest frequency of adverse events (Figure 7B), showed no statistical difference between the two groups (OR = 1.22, 95% CI [0.55, 2.69], Z = 0.48, P = 0.63) and no heterogeneity (I^2^ = 0%, P = 0.56). In summary, TPTD combined with anti-bone-resorption drugs showed no significant difference in total adverse events and risk of hypercalcaemia compared to TPTD monotherapy, and the safety performance was comparable.

#### 3.4.8 Publication offset

Taking safety events as an example, a publication offset analysis of the eight included studies was conducted (Figure 7C). The included studies were heterogeneous in the course of treatment (12 months, 18 months, and ≥24 months) and the type of anti-bone resorption drugs (denosumab and bisphosphonates). In the presentation of the publication offset graph, the scatter distribution did not show a completely symmetrical funnel shape, suggesting a certain degree of publication offset. Given the diversity of treatment course settings and types of anti-bone-resorption drugs in the included studies, this study deeply explored the sources of heterogeneity through subgroup analysis. Stratification by treatment course and type of anti-bone-resorption drugs helped reduce the potential impact of publication bias to some extent, thereby supporting the reliability of the study conclusion.

## 4 Discussion

With the acceleration of the global ageing process, osteoporosis and its associated fractures have become serious public health problems, imposing a huge health and economic burden on patients and society (Johnell and Kanis, 2006; Salari et al., 2021; Xiao et al., 2022; Eastell et al., 2019). Currently, anti-osteoporosis drugs include bisphosphonates, TPTD, and RANKL inhibitors. These drugs improve BMD and reduce fracture risk through different action mechanisms; however, their efficacy and safety vary significantly depending on the treatment regimens (Eastell et al., 2019). In clinical practice, monotherapy is the traditional treatment mode, but its long-term efficacy may be limited (Compston et al., 2019; Leder et al., 2015). Combination therapy aims to exert synergistic effects to enhance efficacy through the simultaneous use of multiple drugs (Anastasilakis et al., 2020; Tsai et al., 2013). However, a systematic summary of existing comparative studies on the efficacy and safety of these two treatment strategies is still lacking (Yuan et al., 2019; Shen et al., 2017; Langdahl et al., 2018), leaving clinicians with uncertainty when selecting appropriate treatment options.

This study systematically reviewed eight RCTs to explore the efficacy, regulation of bone metabolism, and safety of TPTD combined with anti-bone-resorption drugs (bisphosphonates or denosumab) for the treatment of osteoporosis. The results show that combination therapy has significant advantages in improving BMD, but fails to reduce the risk of vertebral and non-vertebral fractures. There are significant differences in the effects of different drug combination strategies on bone turnover markers, which has important guiding significance for clinical decision-making.

### 4.1 Mechanism of the difference in efficacy and clinical implications

BMD growth rate in the lumbar spine, femoral neck, and hip joint of the combined denosumab group was significantly higher than that in the single-drug group, which may be related to the synergistic mechanism of the two drugs. TPTD promotes bone formation by intermittently activating the parathyroid hormone receptors, whereas denosumab reduces bone resorption by inhibiting the RANKL pathway. The combination of the two may form a dynamic balance of “promoting construction and inhibiting dismantling”. The analysis of bone turnover markers further supports this mechanism. Compared with the single-agent group, the inhibition of bone resorption markers (CTX) in the denosumab combination group was longer (57%–65% reduction), while retaining some bone formation activity (OC reduction was 8%–16% lower than that of denosumab alone), suggesting that BMD gain is achieved through targeted regulation of bone remodelling coupling (Leder et al., 2015; Tsai et al., 2013). In contrast, the improvement of BMD in the bisphosphonate combination group was time-dependent: short-term (12–18 months) combination significantly increased BMD, but a 30-month study by Finkelstein et al. showed that long-term combination may lead to a decrease in BMD (MD = -7.70%), which may be related to the excessive inhibition of bone turnover by bisphosphonate long-term combination, reducing the increase in bone formation marker (P1NP) by 60% (107% vs. 199%) compared with the single-drug group, resulting in bone remodelling imbalance (Finkelstein et al., 2010). This phenomenon aligns with the “bone metabolism window” theory, which suggests that the duration of combination therapy should be individually adjusted to avoid bone metabolic decompensation.

### 4.2 Dissociation of fracture risk from BMD improvement

Although combination therapy significantly improved BMD, neither the vertebral nor nonvertebral fracture risk was significantly reduced (OR = 0.93 vs. 0.68, P > 0.3). This separation phenomenon may be due to the following reasons: ① The total number of fracture events included in the study is extremely low (two vertebral bodies/27 non-vertebral bodies), and the statistical efficacy is insufficient. ② BMD growth needs to reach a specific threshold (such as lumbar spine ≥3%) to significantly affect the risk of fracture, and only the denosumab combination group meets this threshold. ③ Dynamic changes in bone turnover markers indicate potential risks. Although bisphosphonate combinations strongly inhibit bone resorption (with CTX reductions of 50%–70%), they may impair the repair ability of bone microstructure due to excessive bone formation inhibition in the long term, as evidenced by the limited increase of P1NP. In contrast, the denosumab combination group may be more conducive to bone quality improvement by moderately inhibiting bone resorption and maintaining bone formation activity (Muschitz et al., 2013). ④ The risk of fracture is affected by multifactorial determinants beyond BMD, particularly bone quality attributes such as trabecular connectivity and microarchitecture integrity. Experimental and clinical studies have established that trabecular bone score (TBS) and cortical porosity independently predict fracture risk, even in individuals achieving BMD thresholds (Samelson et al., 2019; Silva and Leslie, 2017; Lu et al., 2022). For instance, diminished trabecular connection points (>30% reduction) can reduce bone strength by 50% without altering BMD, as demonstrated in biomechanical models (Oftadeh et al., 2015; Liu et al., 2013). This may explain why BMD gains in our analysis did not translate to fracture reduction: combination therapies might inadequately restore compromised bone quality components (e.g., microcrack accumulation or collagen cross-linking defects). Prior studies of anabolic agents similarly observed that TBS improvements lag behind BMD changes by 6–12 months, suggesting bone quality recovery requires prolonged remodeling cycles (Sibai et al., 2011; Cosman, 2014).

Therefore, future efficacy evaluations should integrate advanced imaging modalities (e.g., HR-pQCT for trabecular number and cortical thickness) with BMD measurements (Small, 2005). Our findings align with the “mechanostat” theory, wherein fracture resistance depends on both bone mass (BMD) and structural competence (microarchitecture) (Augat and Schorlemmer, 2006; Dittmer and Firth, 2017).

### 4.3 Clinical application recommendations and safety considerations

①For high-risk patients who need to rapidly improve BMD, such as multiple fractures OR BMD T value <-3.0, TPTD combined with denosumab may be the preferred regimen, and its short-term (12–18 months) efficacy is significant and safe (total adverse events OR = 1.51, P = 0.06; hypercalcaemia OR = 1.22, P = 0.63). Based on changes in bone turnover markers, P1NP and CTX levels should be monitored in clinical practice. If P1NP increase is <100% or the inhibition of CTX is >70%, the risk of excessive inhibition should be considered and treatment course should be adjusted over time. ② In denosumab combination therapy, if the OC reduction is >50% or the CTX inhibition is >65%, it suggests that the bone resorption inhibition is sufficient, and the treatment period can be extended to 24 months (Leder et al., 2015). However, it should be noted that: ① the combination of bisphosphonates for more than 24 months may weaken the efficacy; ② the evidence of male patients is limited (only one study was included), and the conclusion should be extrapolated cautiously; and ③ although the short-term safety appears favourable, the potential long-term risks (>3 years) of combination therapy—such as atypical fractures and jaw osteonecrosis—require further verification.

### 4.4 Research limitations and future directions

The limitations of this study include: ① A restricted number of included studies (n = 8) with small sample sizes (maximum n = 150), potentially compromising result stability. ② Heterogeneity across studies in treatment duration (9–30 months), drug types (bisphosphonate/denosumab), and population characteristics (predominantly female). Although subgroup analyses were implemented, residual confounding bias may persist. ③ Non-standardized reporting of bone turnover markers - including missing baseline values and exclusive use of percentage change metrics - constrains comprehensive analysis of bone metabolism dynamics. ④ Funnel plot asymmetry indicates publication bias, with potential exclusion of negative-result studies. Future investigations should prioritize multicenter, large-scale longitudinal studies (e.g., 5-year follow-up) to examine: ① Combined therapy’s prophylactic efficacy and fracture-risk thresholds; ② Sequential treatment optimization (e.g., transitioning to monotherapy after combination regimens); ③ Personalized protocols guided by bone turnover markers (e.g., P1NP/CTX ratios). Expanded enrollment should incorporate male participants and special populations (e.g., glucocorticoid-induced osteoporosis cases), alongside standardized biomarker reporting (absolute changes with standard deviations) to improve conclusion generalizability.

## 5 Conclusion

This meta-analysis of eight randomized controlled trials demonstrates that TPTD combined with denosumab produces significantly greater improvements in bone mineral density (BMD) at the lumbar spine, femoral neck, and total hip compared to TPTD monotherapy, yet reveals a critical dissociation between BMD gains and fracture risk reduction (vertebral: OR = 0.93 vs. 0.68, P > 0.3). The findings emphasize that while combination therapy exhibits synergistic effects on bone remodeling coupling, BMD assessment alone cannot fully predict anti-fracture efficacy; comprehensive risk evaluation requires integration of bone microstructure analysis and dynamic biomarkers (e.g., P1NP/CTX ratios).

Notably, bisphosphonate combinations showed time-dependent limitations, with long-term use (>24 months) potentially impairing bone formation through excessive turnover suppression. In contrast, denosumab combinations maintained balanced remodeling activity, suggesting better preservation of bone quality despite equivalent fracture outcomes. For high-risk patients requiring rapid BMD elevation, TPTD-denosumab combination remains clinically preferable, provided treatment duration is guided by biomarker thresholds (e.g., CTX inhibition ≤70%, P1NP increase ≥100%).

Future research should prioritize longitudinal studies correlating BMD trajectories with high-resolution microstructural imaging to establish fracture-risk thresholds for combination therapies. Standardized reporting of absolute bone turnover marker changes and extended safety monitoring (>5 years) are essential for optimizing personalized treatment strategies.

## Data Availability

The original contributions presented in the study are included in the article/supplementary material, further inquiries can be directed to the corresponding author.
